# Clinical performance of lung ultrasound in predicting ARDS morphology

**DOI:** 10.1186/s13613-021-00837-1

**Published:** 2021-03-29

**Authors:** Andrea Costamagna, Emanuele Pivetta, Alberto Goffi, Irene Steinberg, Pietro Arina, Anna Teresa Mazzeo, Lorenzo Del Sorbo, Simona Veglia, Ottavio Davini, Luca Brazzi, V. Marco Ranieri, Vito Fanelli

**Affiliations:** 1 Department of Anaesthesia and Critical Care, AOU Città della Salute e della Scienza di Torino, University of Turin, Corso Dogliotti 14, 10126 Turin, Italy; 2 Department of General and Specialized Medicine, Division of Emergency Medicine and High Dependency Unit, Cancer Epidemiology Unit - AOU Città Della Salute e Della Scienza di Torino, Turin, Italy; 3 Interdepartmental Division of Critical Care Medicine and Department of Medicine, University of Toronto, Toronto, ON Canada; 4 Department of Medicine, Division of Critical Care Medicine, St. Michael’s Hospital, Toronto, ON Canada; 5 Department of Surgical Sciences, University of Turin, Turin, Italy; 6 Department of Medicine, Division of Respirology (Critical Care), University Health Network, Toronto, ON Canada; 7 Department of Diagnostic Imaging and Radiotherapy, AOU Città della Salute e della Scienza di Torino–University of Turin, Turin, Italy; 8 Alma Mater Studiorum, Dipartimento di Scienze Mediche e Chirurgiche, Anesthesia and Intensive Care Medicine, Policlinico di Sant’Orsola, Università di Bologna, Bologna, Italy; 9 Dipartimento di Patologia Umana Dell’adulto e Dell’età Evolutiva, Anestesia e Rianimazione, Univesity of Messina, Messina, Italy

**Keywords:** Lung ultrasound, ARDS, ARDS morphology, Bedside tests, Point of care diagnostic tests, Respiratory monitoring

## Abstract

**Background:**

To assess diagnostic performance of lung ultrasound (LUS) in identifying ARDS morphology (focal vs non-focal), compared with the gold standard computed tomography.

**Methods:**

Mechanically ventilated ARDS patients undergoing lung computed tomography and ultrasound were enrolled. Twelve fields, were evaluated. LUS score was graded from 0 (normal) to 3 (consolidation) according to B-lines extent. Total and regional LUS score as the sum of the four ventral (LUS_V_), intermediate (LUS_I_) or dorsal (LUS_D_) fields, were calculated. Based on lung CT, ARDS morphology was defined as (1) focal (loss of aeration with lobar distribution); (2) non-focal (widespread loss of aeration or segmental loss of aeration distribution associated with uneven lung attenuation areas), and diagnostic accuracy of LUS in discriminating ARDS morphology was determined by AU-ROC in training and validation set of patients.

**Results:**

Forty-seven patients with ARDS (25 training set and 22 validation set) were enrolled. LUS_TOT_, LUS_V_ and LUS_I_ but not LUS_D_ score were significantly lower in focal than in non-focal ARDS morphologies (*p* < .01). The AU-ROC curve of LUS_TOT_, LUS_V_, LUS_I_ and LUS_D_ for identification of non-focal ARDS morphology were 0.890, 0.958, 0.884 and 0.421, respectively. LUS_V_ value ≥ 3 had the best predictive value (sensitivity = 0.95, specificity = 1.00) in identifying non-focal ARDS morphology. In the validation set, an LUS_V_ score ≥ 3 confirmed to be highly predictive of non-focal ARDS morphology, with a sensitivity and a specificity of 94% and 100%.

**Conclusions:**

LUS had a valuable performance in distinguishing ARDS morphology.

**Supplementary Information:**

The online version contains supplementary material available at 10.1186/s13613-021-00837-1.

## Background

Acute respiratory distress syndrome (ARDS) is characterized by significant loss of lung aeration and increased lung weight as a consequence of increased lung permeability leading to accumulation of protein-rich edema [1]. Computed tomography (CT) is the gold standard imaging technique for the identification, characterization of distribution, and quantification of loss of lung aeration during ARDS [2]. CT scan thus can predict the potential for alveolar recruitment, which is variable among patients with ARDS [3]. Compared to focal (lobar loss of lung aeration), non-focal (diffuse/patchy loss of lung aeration) pattern show greater alveolar recruitment and less signs of over-distension when an open lung strategy is used (i.e., use of recruitment maneuvers and higher positive end-expiratory pressure) [2]. Lung ultrasound (LUS) has been proposed as an accurate bedside and radiation-free technique for evaluation of lung consolidations [4] and for follow-up of aeration changes in response to interventions [5–7]. Scores based on detection of B-lines (the sonographic sign of increased lung density associated with interstitial syndrome) and on consolidation have been correlated with global and regional lung aeration as assessed by CT [8, 9]. However, the role of LUS in identifying ARDS morphologic pattern has not been investigated. Therefore, our study explored the feasibility and accuracy of lung ultrasound as imaging technique for identification of ARDS morphology (focal vs non-focal) as compared to CT scan. We hypothesized that LUS performed at the bedside accurately quantifies aeration loss in ARDS patients, providing useful information about ARDS morphology (focal vs non-focal).

## Methods

### Subjects

All consecutive mechanically ventilated patients admitted to a tertiary center intensive care unit (ICU) with a diagnosis of ARDS [10], with an expected duration of mechanical ventilation greater than 24 h and undergoing CT scan evaluation of the lung parenchyma, were included. Exclusion criteria were age below 18 years, confirmed diagnosis of pulmonary fibrosis or moribund patient. The local Ethics Committee approved the study protocol (0117126) and written consent was obtained according to Italian regulation.

### Study protocol

All patients underwent to lung CT scan at study entry. Immediately after every CT scan completion, LUS was performed at bedside in the ICU, with the same level of sedation and ventilator settings as during the CT scan.

### Lung ultrasound

**Fig. 1:**
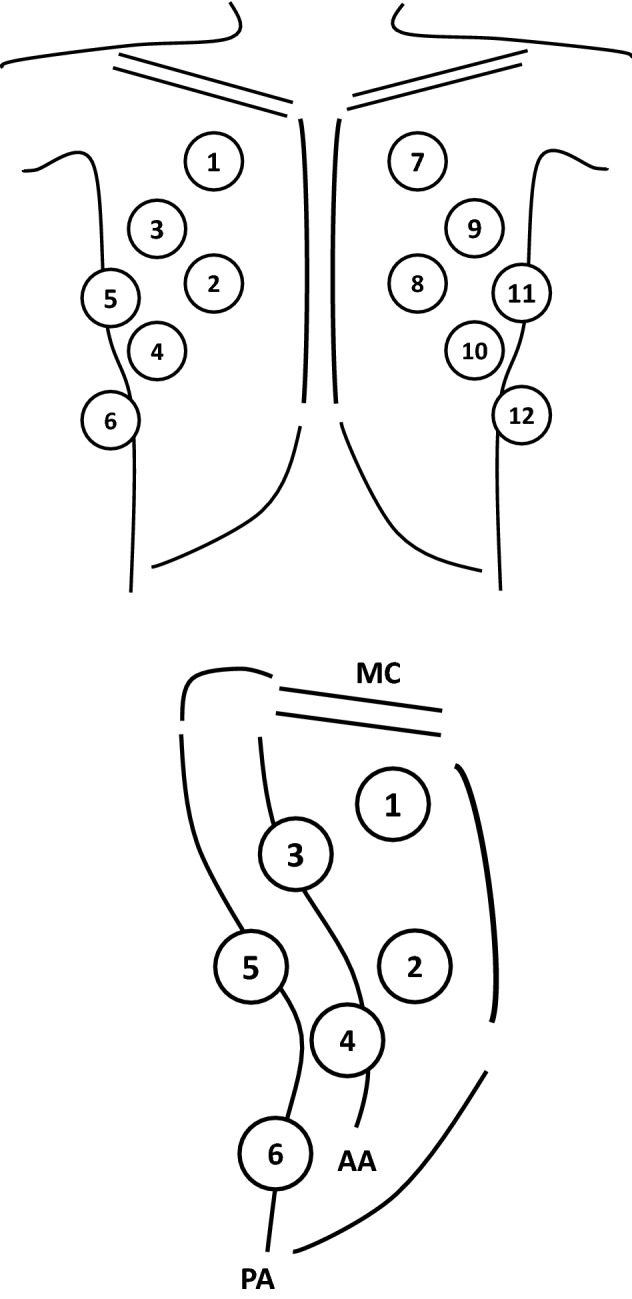
Representative image of ultrasound anatomical landmarks

Patients were examined in supine position, using a portable ultrasound machine (Mylab™ seven, Esaote S.p.A, Genova, Italy) equipped with a curvilinear transducer (5–3 MHz) [11]. Twelve fields, six for each hemithorax, were analyzed based on predefined anatomical landmarks to encompass ventral, intermediate and dorsal lung zones [11, 12]; a detailed description of theselandmarks is given in ESM document (Fig. 1; Additional file 2). Each area was examined for identification of four ultrasound aeration patterns [5, 7, 13–15]: 1) normal aeration (N): presence of lung sliding and/or lung pulse with A-lines or fewer than two isolated B-lines/intercostal space; 2) moderate loss of lung aeration (B1 profile): multiple spaced B-lines, ≥ 3/intercostal space; 3) severe loss of lung aeration (B2 profile): multiple coalescent B lines (± subpleural consolidations); and 4) lung consolidation (C): presence of a tissue pattern ± air bronchograms. For each field of interest, a score was assigned: N = 0, B1 = 1, B2 = 2, C = 3 [13]. A total Lung Ultrasound Score (LUS_TOT_), ranging between 0 and 36, was calculated as the sum of individual scores of each field [13]. Regional Lung Ultrasound Score to assess the effect of gravity on lung aeration was also calculated. LUS score in the ventral lung regions (LUS_V_) was calculated as the sum of the scores of the fields 1, 2, 7 and 8; in the intermediate lung regions (LUS_I_) was the sum of the scores of the fields 3, 4, 9 and 10; in the dorsal lung regions (LUS_D_) was the sum of the scores of the fields 5, 6, 11 and 12. Each regional score ranged from 0 to 12. Inter-observer agreement between operators was evaluated in validation set using Cohen κ with associated 95% confidence intervals.

### Lung computed tomography

Lung CT scans were obtained at study entry. Twelve lung areas, six for each hemithorax, were identified on CT axial plane images using pre-defined anatomical landmarks matching previously described twelve LUS regions of interest. A detailed description of these landmarks is given in ESM document. Quantitative analysis of the previously acquired DICOM files was performed blindly using a dedicated software (Maluna®, University of Mannheim, Germany) [16, 17]. The total area of the selected region of interest consisted of a finite number of pixels. The X-ray attenuation of each pixel, expressed in Hounsfield units (HU), was obtained by determining the percentage of radiation adsorbed [17]. The following hyperinflated (between − 900 and − 1000 HU); normally aerated (− 900 and − 500 HU); poorly aerated (− 500 and − 100 HU); and non-aerated (− 100 and 100 HU) lung compartments were quantified [17].

Two physicians (VF and AC) blindly and independently reviewed and categorized the CT ARDS morphology, according to the CT scan ARDS Study Group criteria [18]. Two ARDS morphologies were defined: (1) focal (loss of aeration with lobar or segmental distribution) and (2) non-focal (widespread loss of aeration or segmental loss of aeration distribution associated with uneven lung attenuation areas—diffuse/patchy).

### Statistics

The study was conducted in two phases. In Phase 1, a first group of patients (**training set**) was analysed to determine diagnostic accuracy of global and regional LUS and threshold values able to best discriminate patients with non-focal ARDS morphology. In Phase 2, a second group of patients (**validation set**) was used to prospectively assess the diagnostic performance of LUS thresholds. Descriptive data are presented as mean and standard deviation (SD) or median and interquartile range (IQR) (continuous variables), and as numbers and percentages (categorical variables), as appropriate. Comparisons were performed using paired or unpaired t-test for continuous parametric variables, the Wilcoxon test for matched non-parametric continuous variables, the Wilcoxon-Mann–Whitney or the Kruskal–Wallis test with Dunn's pairwise or Friedman comparison for unpaired or paired continuous variables, as appropriate. Categorical variables were analyzed with Pearson chi-square test or Fisher’s exact test, as appropriate.

Optimal cut-offs values of regional and total LUS scores in identifying ARDS morphologies were analyzed by non-parametric Receiver Operating Characteristic (ROC) curve analyses with Youden method for empirical cut-point estimation. Statistical analyses were performed using Stata 13.1/SE (Stata Corporation, Texas, USA).

## Results

### Study population

**Fig. 2:**
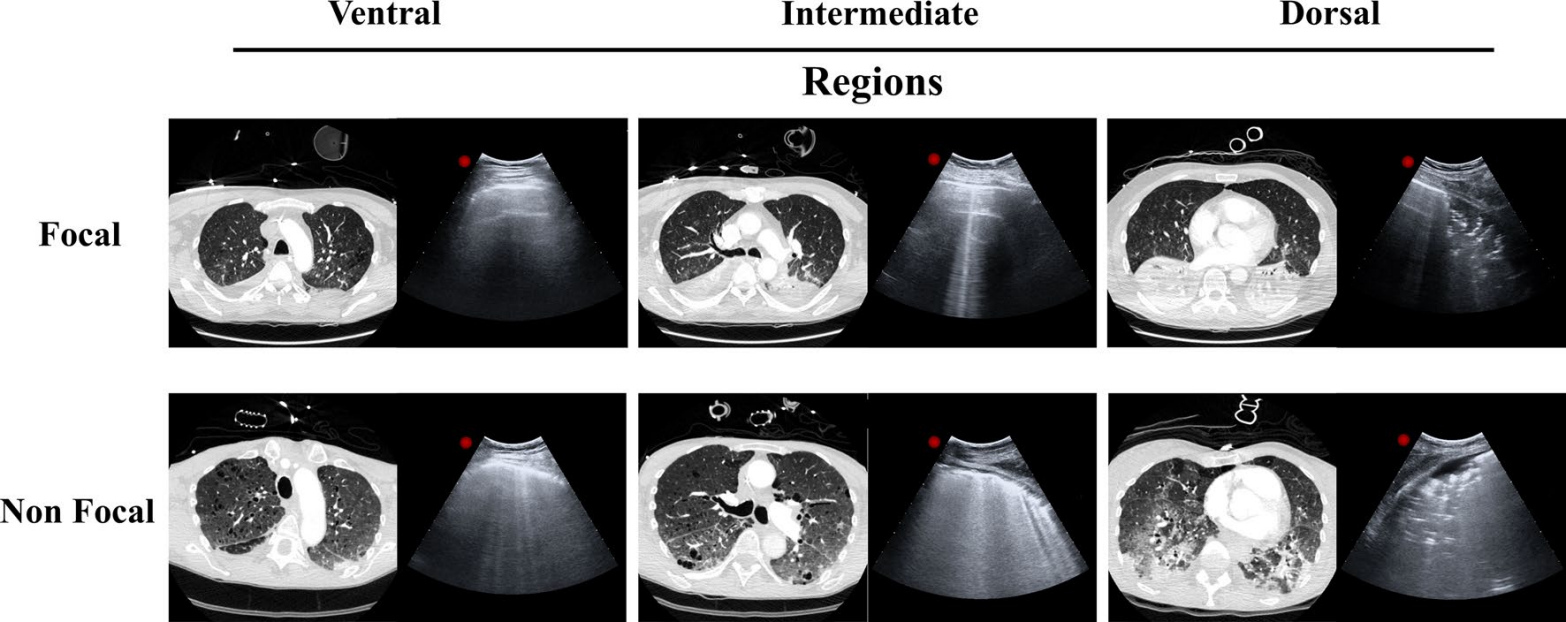
Representative lung TC images and their corresponding LUS images in ventral, intermediate and dorsal lung regions in focal and non-focal ARDS morphologies

Forty-seven consecutive patients (25 in the training set and 22 in the validation set) were enrolled in the study. Baseline characteristics of patients, ventilation settings, blood gas exchange, hemodynamics are reported in Table 1. Forty-seven lung CT scans were performed (25 in the training set and 22 in the validation set); representative lung CT and LUS images of different ARDS morphologies are shown in Fig. 2.

**Table 1:** Baseline characteristics of the study population patients, ventilation settings, blood gas exchange and hemodynamic

Variables	Overall (*N* = 47)	Training set (*N* = 25)	Validation set (*N* = 22)
Age (years)	52 (44–63)	57 (50–65)	48 (41–58)
PBW (kg)	64 (57–71)	65 (57–71)	64 (58–70)
Gender (M/F)	31/16	17/8	14/8
Risk factors for ARDS—*N* (%)			
Pneumonia	42 (89)	24 (85)	21 (96)
Non-pulmonary sepsis	2 (4)	2 (7)	0 (0)
Pancreatitis	3 (6)	2 (7)	1 (4)
SAPS II score	36 (29–45)	34 (27–44)	39 (29–47)
SOFA score	8 (7–11)	8 (7–11)	9 (7–11)
TV/PBW (mL/kg)	6.6 (5.9–7.8)	6.9 (6.0–7.9)	6.4 (5.8–7.5)
PEEPtot (cmH_2_O)	14 (11–15)	13 (10–15)	14 (12–16)
P_plat_ (cmH_2_O)	25 (23–27)	25 (23–27)	25 (20–26)
FiO_2_	0.6 (0.5–0.8)	0.6 (0.5–0.8)	0.5 (0.45–0.7)
Blood gas exchange			
pH	7.41 (7.35–7.46)	7.41 (7.35–7.45)	7.39 (7.35–7.46)
PaO_2_/FiO_2_	166 (109–232)	156 (108–233)	178 (114–240)
PaCO_2_	48 (41–52)	48 (41–52)	48 (42–50)
Lactate	1.7 (1.3–2.2)	1.7 (1.3–2.6)	1.6 (1.2–1.8)
MAP (mmHg)	83 (75–89)	82 (73–88)	84 (77–94)
HR (bpm)	89 (77–98)	91 (81–98)	86 (77–100)

PBW: predicted body weight; TV: tidal volume; RR: respiratory rate; PEEP: positive end-expiratory pressure; P_plat_: plateau pressure; FiO_2_: fraction of inspired oxygen; PaO_2_: partial pressure of oxygen (arterial blood); PaCO_2_: partial pressure of carbon dioxide (arterial blood); MAP: mean arterial pressure; HR: heart rate

### Total and regional LUS score in different ARDS morphologies

**Fig. 3:**
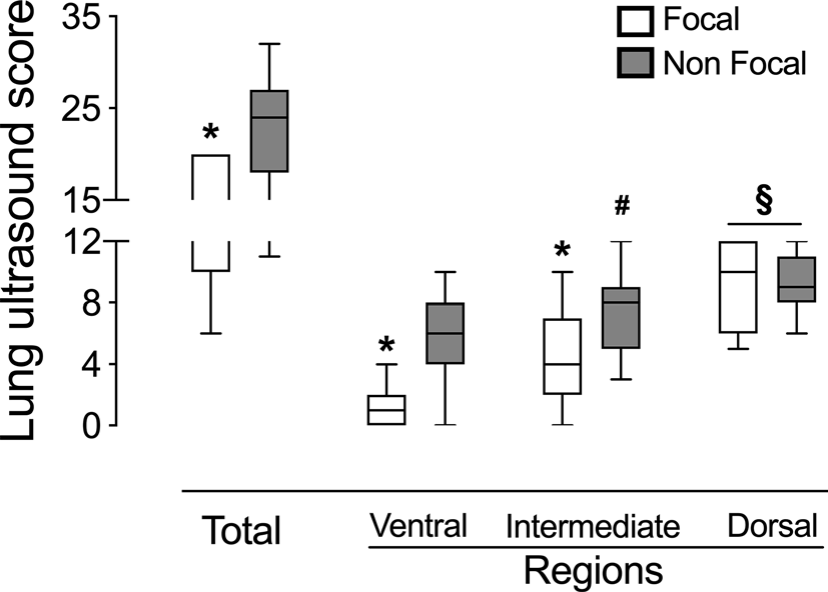
Total and regional LUS score in focal and non-focal ARDS morphologies in the overall population. *p < 0.01 focal vs non-focal ARDS morphologies; ^#^*p* < 0.01 Ventral vs Intermediate lung regions in non-focal ARDS morphology; ^§^*p* < 0.01 Ventral and ^§^*p* < 0.05 Intermediate vs Dorsal lung regions in focal and non-focal ARDS morphology

Overall, LUS_TOT_ was significantly lower in the focal compared to the non-focal ARDS morphology [focal 14 (IQR 10–20); non-focal 24 (IQR 18–27), *p* < 0.01] (Fig. 3). In both ventral and intermediate regions, LUS scores were significantly lower in focal [LUS_V_ 1 (IQR 0–2); LUS_I_ 4 (IQR 2–7) compared to non-focal [LUS_V_ 6 (IQR 6–8), *p* < 0.01; LUS_I_ 8 (IQR 5–9), *p* < 0.05] ARDS morphology (Fig. 3). Finally, in focal morphology, LUS score was significantly lower in ventral compared to dorsal [LUS_V_ 1 (0–2) vs LUS_D_ 10 (6–12); *p* < 0.01] and in intermediate vs dorsal lung regions [LUS_I_ 4 (IQR 2–7) vs LUS_D_ 10 (6–12); *p* < 0.05]. In non-focal morphology, LUS score was significantly lower in ventral compared to intermediate and dorsal lung regions [LUS_V_ 6 (4–8) vs LUS_I_ 8 (5–9); *p* < 0.01 and LUS_D_ 9 (8–11); *p* < 0.01] and in intermediate compared to dorsal regions [LUS_I_ 8 (5–9) vs LUS_D_ 9 (8–11); *p* < 0.01] (Fig. 3). A detailed description of regional and global LUS in training and validation set is presented in Table 2. Inter-observer agreement showed a substantial agreement (κ = 0.87, 95% CI 0.81–0.92).

**Table 2:** Global and regional LUS scores in overall patients and in training and validation sets

Variables	Overall (*N* = 47)		Training set (*N* = 25)		Validation set (*N* = 22)	
	Focal (*N* = 11)	Non-focal (*N* = 36)	Focal (*N* = 5)	Non-focal (*N* = 20)	Focal (*N* = 6)	Non-focal (*N* = 16)
LUS_TOT_	14 (10–20)	24 (18–27)*	14 (8–19)	24 (19–27)*	16 (12–20)	22 (17–27) **
LUS_V_	1 (0–2)	6 (4–8)*	1 (0–1)	6 (5–8)*	2 (1–3)	6 (4–7)*
LUS_I_	4 (2–7)	8 (5–9)*,^o^	3 (1–7)	9 (6–9)**^,oo^	4 (3–9)	8 (4–10)
LUS_D_	10 (6–12)^o,@@^	9 (8–11)^o,@@^	12 (6–12)^oo^	9 (8–11)^o^	10 (7–11)^o^	10 (7–12) ^o^

Abbreviations: LUS_TOT_: lung ultrasound score total; LUS_V_: lung ultrasound score ventral region; LUS_I_ lung ultrasound score intermediate region; LUS_D_ lung ultrasound score dorsal region

^*^p < 0.01, **p < 0.05 VS focal; ^o^*p* < 0.01, ^oo^*p* < 0.05 VS LUS_V_; ^@@^ *p* < 0.05 VS LUS_I_

### Amount of normally, poorly and not aerated lung tissue at different LUS scores

To better understand the differences between LUS and CT in assessing lung aeration, we analyzed the distribution of lung aeration on CT at different LUS score (Additional file 1: Fig. S1). In both focal and non-focal ARDS morphologies, the amount of normally aerated tissue significantly decreased from LUS 0 to 3, while the amount of not aerated tissue significantly increased (Additional file 1: Fig. S1 panel A and B). On the contrary, the amount of poorly aerated tissue did not change at different scores (Additional file 1: Fig. S1 panel A) in focal ARDS while significantly increased in non-focal ARDS (Additional file 1: Fig. S1 panel B, Additional file 2).

### Accuracy of LUS in identifying ARDS morphology

**Fig. 4:**
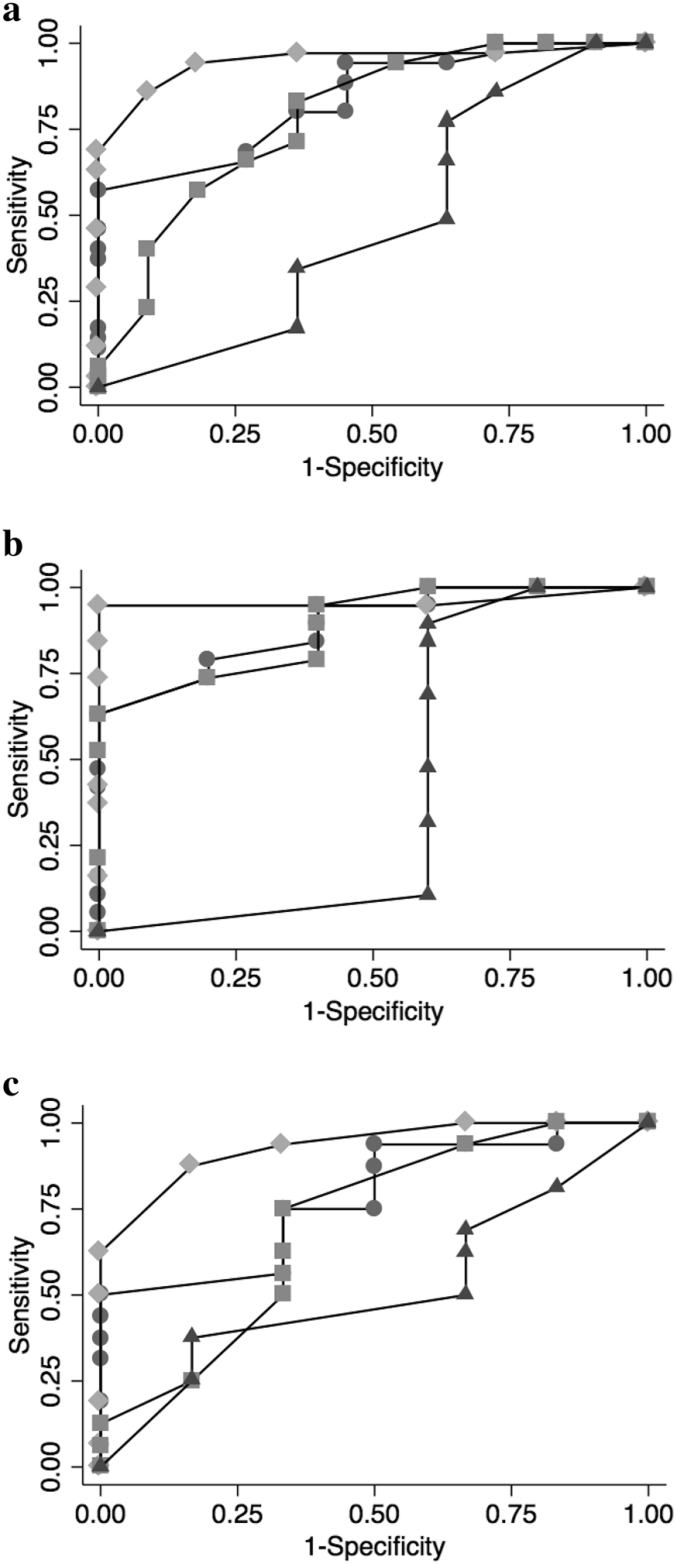
Combined Receiver Operating Characteristic (ROC) curves of overall (panel A), training set (panel B) and validation set (panel C) for total (circles) and regional ventral (rhombus), intermediate (squares) and dorsal (triangles) LUS score in identifying non-focal ARDS morphologies. AUC_ROC_ (95% CI) of LUS_V_ for non-focal ARDS was 0.948 (0.888–1.000), 0.958 (0.881–1.000) and 0.932 (0.832–1.000) in overall, training set and validation set, respectively

In the overall population, the areas under the ROC curve of LUS_TOT_, LUS_V_, LUS_I_ and LUS_D_ for identification of non-focal ARDS morphology were 0.839, 0.948, 0.786 and 0.478, respectively. In the training set, the areas under the ROC curve of LUS_TOT_, LUS_V_, LUS_I_ and LUS_D_ for identification of non-focal ARDS morphology were 0.890, 0.958, 0.884 and 0.421, respectively. In the validation set, the areas under the ROC curve of LUS_TOT_, LUS_V_, LUS_I_ and LUS_D_ for identification of non-focal ARDS morphology were 0.781, 0.932, 0.703 and 0.516, respectively (Fig. 4). An LUS_V_ score ≥ 3 (calculated on training set) had the best predictive value (sensitivity = 0.95, specificity = 1.00) for the identification of non-focal ARDS morphology (Table 3). In the validation set, an LUS_V_ score ≥ 3 confirmed to be highly predictive of non-focal ARDS morphology, with a sensitivity and a specificity of 94% (95% CI 70–100%) and 100% (95% CI 54–100%), respectively, and a positive predictive value and a negative predictive value of 100% and 86% (95% CI 47–98%), respectively.

**Table 3:** Accuracy of prediction of regional LUS_V_ score for non-focal ARDS morphology, by non-parametric receiver operating characteristic (ROC) analysis in the training set

LUS_V_	Sensitivity (%)	Specificity (%)	Correctly classified (%)	LR+	LR−
≥ 0	100.00	0.00	80.00	1.0000	
≥ 1	95.00	40.00	84.00	1.5833	0.1250
≥ 3	95.00	100.00	96.00		0.0500
≥ 4	85.00	100.00	88.00		0.1500
≥ 6	75.00	100.00	80.00		0.2500
≥ 7	40.00	100.00	52.00		0.6000
≥ 8	35.00	100.00	48.00		0.6500
≥ 9	15.00	100.00	32.00		0.8500
> 9	0.00	100.00	20.00		1.0000
Observations (*n*)	25, training set				
ROC area (SE; 95% CI)	0.9600 (0.0405; 0.88069–1.0000)				

LUS: lung ultrasound score; LR: likelihood ratio; ROC: receiver-operating characteristics curve

## Discussion

The main finding of this study is that LUS score ≥ 3 in the ventral lung regions accurately excludes focal ARDS morphology.

Recently, it has been shown that LUS identifies patients with pulmonary edema [4] at risk of developing ARDS [8, 19, 20], especially if mechanically ventilated [21], and it notably correlates with changes in lung tissue aeration [9]. Our data showed that LUS reliably identifies ARDS morphology. In fact, exploring only four fields of non-dependent (ventral) lung regions, LUS score equal or higher than 3 accurately excludes lobar ARDS. This LUS application is clinical relevant, because patients with focal lung morphology at ZEEP are at risk of significant hyperinflation of their baby lung during recruitment maneuvers and the extent of lung recruitment is quite limited [2]. On the contrary, patients with diffuse aeration loss may benefit from recruitment maneuvers and higher PEEP levels especially in presence of life-threatening hypoxemia [22]. Bouhemad and colleagues showed a good correlation between pressure–volume curves and LUS assessment of PEEP induced recruitment. However, this association may imply over-inflation of well aerated regions [7]. In fact, mechanical ventilation with open lung strategy may not result in a homogeneous lung parenchyma, as recruited lung does not always reassume the elastic characteristics of normally aerated lung [23], thereby increasing the risk of hyperinflation of normally aerated alveoli. Moreover, Chiumello and colleague demonstrated that changes in LUS have not been associated with alveolar recruitment as demonstrated by lung CT analysis when the level of PEEP was increased from 5 to 15 cmH2O [9]. In fact, PEEP related changes of global LUS weakly correlated with lung CT decrease of not aerated tissue [9]. Our data may in part explain why changes in LUS score were not able to assess positive end expiratory pressure induced lung recruitment [9]. In fact, in focal ARDS morphology increasing positive end expiratory pressure from 5 to 15 may induce over-inflation of already open alveolar units without recruitment. Our findings allow us to speculate that LUS evaluation, confined to only four ventral lung regions, can help the clinician to evaluate the ARDS morphology at the bedside and be part of pre- and post-test probability to predict response to PEEP and lung recruitment maneuvers [24]. In fact, balancing risk and benefit of higher levels of PEEP in individualized cases is warranted [25]: Recently, the Lung Imaging for Ventilator Setting in ARDS (LIVE) study failed to demonstrate 90-day improvement mortality of ARDS patients who underwent personalized mechanical ventilation strategy based on radiographic phenotype (focal vs non-focal). However, 21% of the radiographic phenotypes were misclassified and only 34% of patients were actually classified using CT scans, with the remainder classified using chest X-ray [26]. In light of these results, the role of LUS in phenotyping ARDS patients need to be addressed in future clinical trials [27].

Some limitations of the current study should be addressed. First, different spatial resolution of CT and LUS may affect the evaluation of lung aeration. In fact, different from LUS, CT scan analysis encompasses the total area of interest along the pleural side of the chest wall and the inner boundary along the mediastinal organs. Second, the current study was performed at single center, then further external validation of identified cutoff to distinguish between lobar from diffuse/patchy ARDS morphology is warranted. Third, the current study design was observational; future studies are needed to evaluate response to PEEP based on ARDS morphology as defined by LUS. Finally, this study was performed before the Covid-19 pandemic and our cohort did not include patients with Covid-19 associated ARDS (CARDS). We speculate that our findings may apply also to CARDS patients; however, further studies are needed to address this issue.

## Conclusions

In this cohort of patients with ARDS, LUS was a reliable bedside tool able to distinguish focal from non-focal morphologies. Using LUS in terms of pre-test probability to set ventilation strategy in individualized cases should be investigated further.

## Supplementary Information

**Additional file 1: Figure S1.** Percentage of normally (blue boxes), poorly (red boxes) and not aerated (grey boxes) lung tissue at different LUS scores in Focal (panels A), and Non Focal (panel B) ARDS morphologies. *p<.05 Normal aerated tissue at LUS 0 vs 1, 2 and 3. #p<0.05 Poorly aerated tissue at LUS 0 vs 1, 2, and 3. °p<0.05 Not aerated tissue at LUS 0 vs 1, 2 and 3. Blu, red and grey box plots indicate normally, poorly and not aerated lung tissue.

**Additional file 2.** Supplementary material and methods and results.

## Data Availability

The datasets generated and/or analysed during the current study are not publicly available due to ethic and privacy statements but are available from the corresponding author on reasonable request.

## Abbreviations

ARDS: Acute respiratory distress syndrome
CT: Computed tomography
LUS: Lung ultrasound
ICU: Intensive care unit
LUS_TOT_: Total Lung Ultrasound Score
LUS_V_: LUS score in the ventral lung regions
LUS_I_: LUS score in the intermediate lung regions
LUS_D_: LUS score in the dorsal lung regions
HU: Hounsfield units
P_air_: Percentage of aerated tissue in regions of interest
ROC: Receiver Operating Characteristic
