# Supplementary material for: Clinical performance of lung ultrasound in predicting ARDS morphology
Source: Ann Intensive Care. 2021 Mar 29;11:51. doi: 10.1186/s13613-021-00837-1 (PMC8006629; doi:10.1186/s13613-021-00837-1)
Supplement: Supplementary file 2 — Additional file 2. Supplementary material and methods and results. [file 13613_2021_837_MOESM2_ESM.docx]

**Clinical Performance of Lung Ultrasound in Predicting ARDS Morphology**

**ELECTRONIC SUPPLEMENTARY MATERIAL**

Costamagna Andrea^1^, Pivetta Emanuele ^2^, Goffi Alberto^3,4^ , Steinberg Irene^5^, Arina Pietro^5^, Mazzeo Anna Teresa ^5,9^, Del Sorbo Lorenzo^3,6^, Veglia Simona ^7^, Davini Ottavio ^7^, Brazzi Luca^1,5^, Ranieri V Marco ^8^, Fanelli Vito * ^1,5^

^1^ Department of Anaesthesia and Critical Care - AOU Città della Salute e della Scienza di Torino – University of Turin, Italy

^2^ Department of general And Specialized Medicine, Division of Emergency Medicine and High Dependency Unit, Cancer Epidemiology Unit - AOU Città della Salute e della Scienza di Torino – Turin, Italy

^3^ Interdepartmental Division of Critical Care Medicine and Department of Medicine, University of Toronto, Toronto, ON, Canada

^4^ Department of Medicine, Division of Critical Care Medicine, St. Michael’s Hospital, Toronto, ON, Canada

^5^ Department of Surgical Sciences, University of Turin, Italy^6^ Department of Medicine, Division of Respirology (Critical Care), University Health Network, Toronto, ON, Canada

^7^ Department of Diagnostic Imaging and Radiotherapy - AOU Città della Salute e della Scienza di Torino – University of Turin, Italy

^8^ Alma Mater Studiorum - Università di Bologna, Dipartimento di Scienze Mediche e Chirurgiche, Anesthesia and Intensive Care Medicine, Policlinico di Sant'Orsola, Bologna, Italy.

^9.^ Dipartimento di Patologia Umana dell'adulto e dell'età evolutiva. Anestesia e rianimazione. Univesity of Messina, Italy

**Corresponding Author:**

* Vito Fanelli MD, PhD

Department of Surgical Sciences, University of Turin, Italy

Anesthesia and Critical Care Medicine - AOU Città della Salute e della Scienza di Torino

University of Turin, Italy

Corso Dogliotti 14, 10126 Torino, Italy.

Tel +39-011 633 4005 (office)

Fax +39-011 6960448

vito.fanelli@unito.it

**Methods**

**Subjects**

We conducted a single center, prospective, observational study in a tertiary center intensive care unit (ICU) (Città della Salute e della Scienza di Torino – Molinette University Hospital, Turin, Italy). We considered eligible all mechanically ventilated patients admitted with a diagnosis of ARDS according to the Berlin definition (1), with an expected duration of mechanical ventilation greater than 24 hours, and undergoing CT scan evaluation of the lung parenchyma. Exclusion criteria were age below 18 years, confirmed diagnosis of pulmonary fibrosis or moribund patient. The local Ethics Committee approved the study protocol (0117126) and written consent was obtained according to Italian regulation.

**Study Protocol**

All patients were ventilated according to the ARDSnet lower tidal volumes protective ventilation strategy and underwent to lung CT scan at study entry. Immediately after every CT scan completion, LUS was performed at the bedside in the ICU, with the patient maintained with the same level of sedation and ventilatory settings as during the CT scan.

**Lung Ultrasound**

Patients were examined in supine position with 30 degrees bed head elevation, using a portable ultrasound system (Mylab^TM^ seven, Esaote S.p.A, Genova, Italy) equipped with a curvilinear transducer (5-3 MHz), as previously described (2). The ultrasound beam was directed transversally along the intercostal space, to insonate the majority of the corresponding lung region as seen on an axial plane (3). Twelve fields, six for each hemithorax, were identified based on the following anatomical landmarks (2, 3) (**Figure 1**): 2^nd^-3^rd^ right and left intercostal (IC) spaces along midclavicular (MC) line (zones 1 and 7, respectively); 5^th^-6^th^ right and left IC spaces along the MC line (zones 2 and 8); 3^rd^-4^th^ right and left IC space along the anterior axillary (AA) line (zones 3 and 9); 6^th^-7^th^ right and left IC space along the AA line (zones 4 and 10); 4^th^- 5^th^ right and left IC space along the posterior axillary (PA) line (zones 5 and 11); 7^th^-8^th^ right and left IC space along the PA line and over the diaphragm (zones 6 and 12). Each area was examined for identification of four previously described ultrasound aeration patterns (4-7): 1) Normal aeration (N): presence of lung sliding and/or lung pulse with A-lines or fewer than two isolated B-lines/intercostal space; 2) Moderate loss of lung aeration (B1 profile): multiple spaced B-lines, ≥ 3/intercostal space; 3) Severe loss of lung aeration (B2 profile): multiple coalescent B lines (+/- subpleural consolidations); 4) Lung consolidation (C): presence of a tissue pattern +/- air bronchograms. For each field of interest, a score was assigned: N = 0, B1 = 1, B2 = 2, C = 3 (6). A total Lung Ultrasound Score (LUS_TOT_), ranging between 0 and 36, was calculated as the sum of individual scores of each field (6). Regional Lung Ultrasound Score to assess the effect of gravity on lung aeration was also calculated. LUS score in the ventral lung regions (LUS_V_) was calculated as the sum of the scores of the fields 1, 2, 7 and 8; in the intermediate lung regions (LUS_I_) was the sum of the scores of the fields 3, 4, 9 and 10; in the dorsal lung regions (LUS_D_) was the sum of the scores of the fields 5, 6, 11 and 12. Each regional score ranged from 0 to 12.

**Lung CT**

Lung CT scans were obtained at study entry. Twelve lung areas, six for each hemithorax, were identified on CT axial plane images using pre-defined anatomical landmarks matching previously described twelve LUS regions of interest. Zones 1 and 7 were identified at the level of the apex of the sternal manubrium, next to the clavicle; zones 2 and 8 at the level of the pulmonary trunk, 2 cm underneath the tracheal bifurcation, at the apex of the sternal body; zones 3 and 9 at the level of the beginning of the lower third of the sternal manubrium, 2 cm below zones 1 and 7, in relation to the aortic arch and the body of the scapula; zones 4 and 10 at the level of the middle third of the sternal body, at the base of the heart; zones 5 and 11 at the level of the lower third of the sternal manubrium, in relation to the tracheal bifurcation; zones 6 and 12 at 2 cm over the diaphragm. Quantitative analysis of the previously acquired DICOM files was performed blindly using a dedicated software (Maluna^®^, University of Mannheim, Germany), as previously described (8, 9). Briefly, lung CT regions of interest were chosen by manually drawing the outer boundary along the pleural side of the chest wall and the inner boundary along the mediastinal organs. CT lung areas were selected manually according to previously described landmarks and named from 1 to 12 following corresponding LUS regions. The total area of the selected region of interest consisted of a finite number of pixels, with each pixel representing a 0.4 mm by 0.4 mm square. The X-ray attenuation of each pixel, expressed in Hounsfield units (HU), was obtained by determining the percentage of radiation adsorbed (9). We identified the following lung compartments: hyperinflated (between -900 and -1000 HU); normally aerated (-900 and -500 HU); poorly aerated (-500 and -100 HU); and non-aerated (-100 and 100 HU). Total area of each compartment was measured as pixel area (0.16 mm^2^) multiplied by the number of pixels in the compartment (9).

Two physicians (VF and AC) blindly and independently reviewed and categorized the CT scans according to ARDS morphology, using the CT scan ARDS Study Group criteria (10). Two ARDS morphologies were defined: 1) focal (loss of aeration with lobar or segmental distribution); 2) non focal (widespread loss of aeration or segmental loss of aeration distribution associated with uneven lung attenuation areas).

**Statistics**

The study was conducted in two phases. In Phase 1, a first group of patients (**training set**) was analysed to determine diagnostic accuracy of global and regional LUS and threshold values able to best discriminate patients with non focal ARDS morphology. In Phase 2, a second group of patients (**validation set**) was used to prospectively assess the diagnostic performance of LUS thresholds.

Descriptive data are presented as mean and standard deviation (SD) or median and interquartile range (IQR) (continuous variables), and as numbers and percentages (categorical variables), as appropriate. Comparisons were performed using paired or unpaired t-test for continuous parametric variables, the Wilcoxon test for matched non-parametric continuous variables, the Wilcoxon-Mann-Whitney or the Kruskal-Wallis test with Dunn's pairwise or Friedman comparison for unpaired or paired continuous variables, as appropriate. Categorical variables were analyzed with Pearson chi-square test or Fisher’s exact test, as appropriate.

The optimal cut-offs values of regional and total LUS scores in identifying ARDS morphologies were analyzed by non-parametric Receiver Operating Characteristic (ROC) curve analyses with Youden method for empirical cut-point estimation.

Statistical significance was defined as p<0.05. Statistical analyses were performed using Stata 13.1/SE (Stata Corporation, Texas, USA).

**Results**

**Study population**

Forty-seven consecutive patients (25 in the training set and 22 in the validation set) were enrolled in the study. Baseline characteristics of patients, ventilation settings, blood gas exchange and hemodynamics are reported in **Table 1**.

Forty-seven lung CT scans were performed (25 in the training set and 22 in the validation set); Focal and non focal ARDS morphologies were present in 5 (20%) and 20 (80%) CT scans in the training set, and in 6 (27%) and 16 (73%) CT scans in the validation set, respectively. Representative lung CT and LUS images of different ARDS morphologies are shown in **Figure 1**.

**Multilevel Regression Model**

Independent variables potentially related to lung aeration such as LUS score, ARDS morphology and gravitational effect were included in a multilevel regression model with percentage of lung aeration, assessed with CT scan, as dependent variable. The results of multilevel regression models are shown in **Table 1S**. The unconditional (null) model predicted an overall mean percentage of aeration of 45.2 (95% CI 40.7 -49.8) with a between-subject SD of 13.4% (95% CI 10.4 –17.2), a within- subject SD of 14.0% (95% CI 13.1–15.0) and an intra-class correlation coefficient (ICC) of 0.48 (95% CI 0.35–0.61). Inclusion of level 1 covariates (LUS score) changed the estimations as follows: LUS score 1 decreased aeration by score 1: -9.4% (95% CI -14.1 - -4.6), score 2 by 10.5% (95% CI -14.8 - -6.3) and score 3 by 16.0% (95% CI -20.5 - -11.6), p<0.01. By including level 2 covariates (i.e. ARDS morphology and gravitational effect), aeration decreased by 8.9% (95% CI -21.2 – 3.4) in focal ARDS. Aeration decreased by 1.8% (95% CI -5.0 - -1.3) and 2.0% (95% CI -5.5 - 1.4) in intermediate and dorsal lung areas, respectively, as compared to ventral areas. A trend in decreased aeration is present with the increase in LUS score. Inclusion of these covariates into the model yielded a between-subject SD of 12.4 (95% CI 9.6 – 15.9), a within- subject SD 13.3 (95% CI 12.4 – 14.2) and an ICC of 0.47 (95% CI 0.34 – 0.60).

**TABLES**

**Table 1S.** Multilevel regression model involving independent variables related to lung aeration

|  | Empty model | Level 1 model | Level 2 model |
| --- | --- | --- | --- |
| Percentage of aeration | 45.2  (95% CI 40.7 -49.8) | 55.0  (95% CI 49.7 – 60.4) | 63.2  (95% CI 51.6 – 74.8) |
| LUS score  (baseline: 0) | - | score 1: -9.4*  (95% CI -14.1 - -4.6)  score 2: -10.5*  (95% CI -14.8 - -6.3)  score 3: -16.0*  (95% CI -20.5 - -11.6) | score 1: -8.9*  (95% CI -13.7 - -4.2)  score 2: -9.4*  (95% CI -13.9 - -5.0)  score 3: -14.6*  (95% CI -19.5 - -9.7) |
| ARDS morphology  (baseline: focal ARDS) | - | - | non focal: -8.9  (95% CI -21.2 – 3.4) |
| Gravity Dependent Effect  (baseline: Ventral areas) | - | - | Intermediate area -1.8  (95% CI -5.0 - -1.3)  Dorsal areas -2.0  (95% CI -5.5 - 1.4) |

* p<0.01 vs baseline

**References**

1. Force ADT, Ranieri VM, Rubenfeld GD, Thompson BT, Ferguson ND, Caldwell E, et al. Acute respiratory distress syndrome: the Berlin Definition. JAMA. 2012;307(23):2526-33.

2. Volpicelli G, Elbarbary M, Blaivas M, Lichtenstein DA, Mathis G, Kirkpatrick AW, et al. International evidence-based recommendations for point-of-care lung ultrasound. Intensive Care Med. 2012;38(4):577-91.

3. Mongodi S, Bouhemad B, Orlando A, Stella A, Tavazzi G, Via G, et al. Modified Lung Ultrasound Score for Assessing and Monitoring Pulmonary Aeration. Ultraschall Med. 2017;38(5):530-7.

4. Bouhemad B, Brisson H, Le-Guen M, Arbelot C, Lu Q, Rouby JJ. Bedside ultrasound assessment of positive end-expiratory pressure-induced lung recruitment. Am J Respir Crit Care Med. 2011;183(3):341-7.

5. Bouhemad B, Liu ZH, Arbelot C, Zhang M, Ferarri F, Le-Guen M, et al. Ultrasound assessment of antibiotic-induced pulmonary reaeration in ventilator-associated pneumonia. Crit Care Med. 2010;38(1):84-92.

6. Soummer A, Perbet S, Brisson H, Arbelot C, Constantin JM, Lu Q, et al. Ultrasound assessment of lung aeration loss during a successful weaning trial predicts postextubation distress*. Crit Care Med. 2012;40(7):2064-72.

7. Lichtenstein DA, Meziere GA, Lagoueyte JF, Biderman P, Goldstein I, Gepner A. A-lines and B-lines: lung ultrasound as a bedside tool for predicting pulmonary artery occlusion pressure in the critically ill. Chest. 2009;136(4):1014-20.

8. Terragni PP, Rosboch G, Tealdi A, Corno E, Menaldo E, Davini O, et al. Tidal hyperinflation during low tidal volume ventilation in acute respiratory distress syndrome. Am J Respir Crit Care Med. 2007;175(2):160-6.

9. Grasso S, Terragni P, Mascia L, Fanelli V, Quintel M, Herrmann P, et al. Airway pressure-time curve profile (stress index) detects tidal recruitment/hyperinflation in experimental acute lung injury. Crit Care Med. 2004;32(4):1018-27.

10. Puybasset L, Cluzel P, Gusman P, Grenier P, Preteux F, Rouby JJ. Regional distribution of gas and tissue in acute respiratory distress syndrome. I. Consequences for lung morphology. CT Scan ARDS Study Group. Intensive Care Med. 2000;26(7):857-69.

**Figures Legend**

**Figure 1S.** Percentage of normally (blue boxes), poorly (red boxes) and not aerated (grey boxes) lung tissue at different LUS scores in Focal (panels A), and Non Focal (panel B) ARDS morphologies. *p<.05 Normal aerated tissue at LUS 0 vs 1, 2 and 3. #p<0.05 Poorly aerated tissue at LUS 0 vs 1, 2, and 3. °p<0.05 Not aerated tissue at LUS 0 vs 1, 2 and 3. Blu, red and grey box plots indicate normally, poorly and not aerated lung tissue.
